# Comparative Proteomic Analysis of *Rana chensinensis* Oviduct

**DOI:** 10.3390/molecules23061384

**Published:** 2018-06-08

**Authors:** Hang Su, He Zhang, Xinghua Wei, Daian Pan, Li Jing, Daqing Zhao, Yu Zhao, Bin Qi

**Affiliations:** 1 Practice Innovations Center, Changchun University of Chinese Medicine, Changchun 130117, China; suhang0720@live.cn (H.S.); prettygirl0122@163.com (L.J.); 2 School of Clinical Medicine, Changchun University of Chinese Medicine, Changchun 130117, China; 18744030771@163.com (D.P.); 3 Jilin Science Service Center, Changchun 130021, China; v_stars@163.com; 4 Jilin Ginseng Academy, Changchun University of Chinese Medicine, Changchun 130117, China; cnzhaodaqing@126.com (D.Z.); yuzhao2016@163.com (Y.Z.); 5 College of Pharmacy, Changchun University of Chinese Medicine, Changchun 130117, China

**Keywords:** iTRAQ, proteomics, *Rana chensinensis* oviduct, regulation, differentially expressed proteins

## Abstract

As one of most important traditional Chinese medicine resources, the oviduct of female *Rana chensinensis* (Chinese brown frog) was widely used in the treatment of asthenia after sickness or delivery, deficiency in vigor, palpitation, and insomnia. Unlike other vertebrates, the oviduct of *Rana chensinensis* oviduct significantly expands during prehibernation, in contrast to the breeding period. To explain this phenomenon at the molecular level, the protein expression profiles of *Rana chensinensis* oviduct during the breeding period and prehibernation were observed using isobaric tags for relative and absolute quantitation (iTRAQ) technique. Then, all identified proteins were used to obtain gene ontology (GO) annotation. Ultimately, KEGG (Kyoto Encyclopedia of Genes and Genomes) enrichment analysis was performed to predict the pathway on differentially expressed proteins (DEPs). A total of 4479 proteins were identified, and 312 of them presented different expression profiling between prehibernation and breeding period. Compared with prehibernation group, 86 proteins were upregulated, and 226 proteins were downregulated in breeding period. After KEGG enrichment analysis, 163 DEPs were involved in 6 pathways, which were lysosome, RNA transport, glycosaminoglycan degradation, extracellular matrix (ECM)–receptor interaction, metabolic pathways and focal adhesion. This is the first report on the protein profiling of *Rana chensinensis* oviduct during the breeding period and prehibernation. Results show that this distinctive physiological phenomenon of *Rana chensinensis* oviduct was mainly involved in ECM–receptor interaction, metabolic pathways, and focal adhesion.

## 1. Introduction

The oviduct is a reproductive organ of females, which plays several important roles in the events related to fertilization and embryo development. The oviduct is not only a passive channel for sperm and eggs transport, but is also a highly active secretory organ, such as estrous cycle and ovulation [1]. It provides the most efficient environment for the success of fertilization and early embryo development [2,3]. The component of oviduct of *Rana chensinensis* is rich in glands, including glycoprotein (such as mucins, collagen, enzymes, and hormones, etc.) and lipoprotein [4]. The dry oviduct of female *Rana chensinensis*, which is recorded in the Chinese Pharmacopoeia, possesses the function of improving immune system and lung function [5].

The hibernation for *Rana chensinensis* ranges from October to February next year, followed by the breeding period from February to June. After the breeding period, *Rana chensinensis* goes into the prehibernation period until October [6]. Distinguishing from the oviduct expansion during the breeding period in other species, a unique physiological phenomenon of *Rana chensinensis* is that its oviduct starts to expand after breeding, reaching a peak by October during the prehibernation [7]. For this reason, *Rana chensinensis* oviduct, as traditional Chinese medicine, was collected from frog in the autumn before hibernation. To ascertain the signaling pathways involved in the timing of oviduct expansion, we should clarify the changes of macromolecular components between prehibernation and breeding period. Proteomics is an efficient methodology to analyze protein expression, and it will disclose protein expression profiles in different physiological phases [8]. It is widely used to tackle biological problems, whereby the raw data obtained from proteomics is studied further by bioinformatic methodologies [8]. Isobaric tags for relative and absolute quantitation (iTRAQ) is an isobaric labeling method applied in quantitative proteomics by tandem mass spectrometry to identify the amount of proteins from different samples in a single experiment [9]. iTRAQ can separate and identify a variety of proteins, including membrane proteins, proteins of high molecular weight, insoluble proteins, acidic proteins, and alkaline proteins [10].

## 2. Results

### 2.1. Protein Identified through Itraq Technology

**Figure 1:**
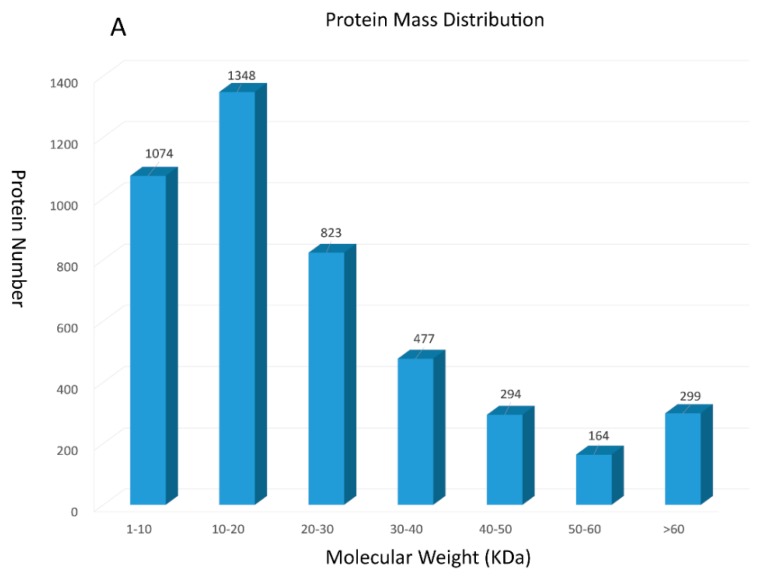

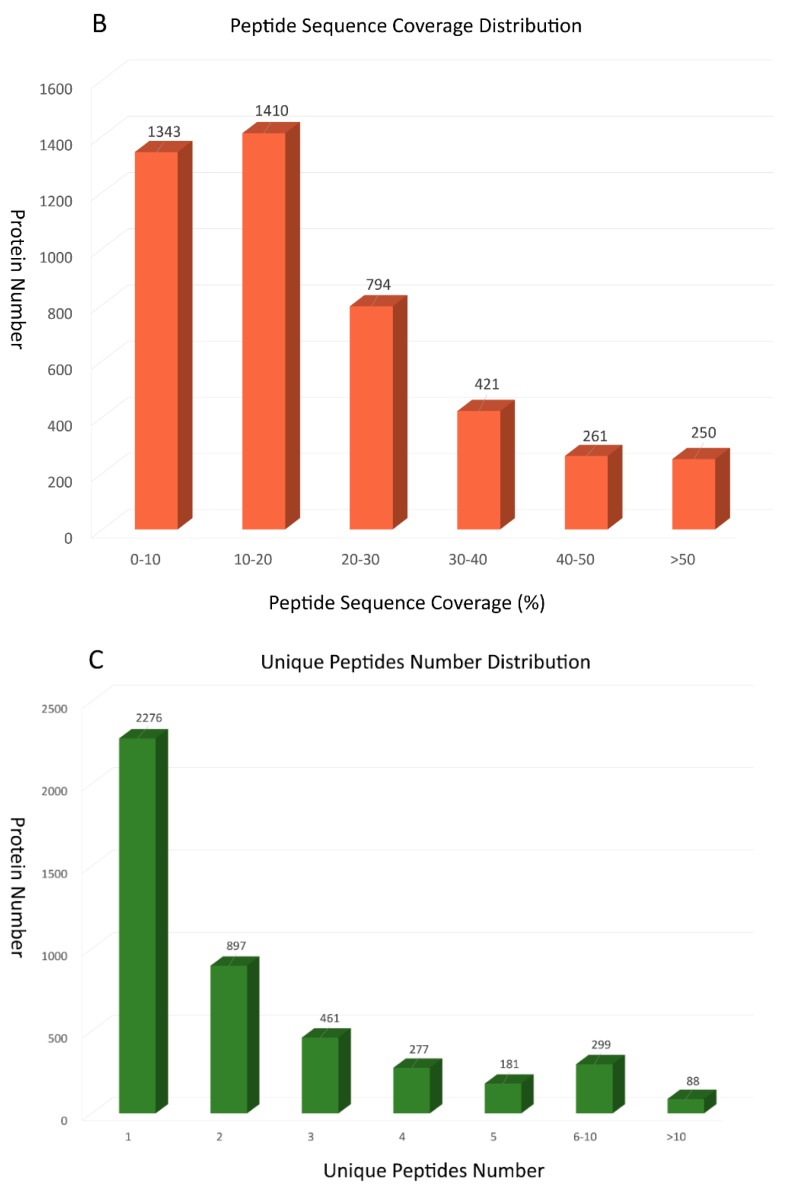
Summary for iTRAQ data: (**A**) The protein mass distribution shown as a histogram; (**B**) The peptide sequence coverage distribution shown as a histogram; (**C**) The number of unique peptides distribution shown as a histogram.

13216 unique peptides and 4479 proteins were identified using the iTRAQ technology (see Tables S1 and S2 in Supplementary Material). Among these identified proteins, 2422 were 0–20 kDa, 1758 were 20–60 kDa, and 299 were over 60 kDa (Figure 1A). Accordingly, we found that identified proteins were primarily below 20 kDa. To further prove the credibility of protein identification, peptide sequence coverage and the unique peptide numbers of proteins were also regarded as two important quality evaluation parameters. Figure 1B showed that peptide sequence coverage of these proteins was basically less than 30%. The number of unique peptides for identified proteins were mainly concentrated in 1 and 2, which makes up approximately 71% of the total unique peptides (Figure 1C).

### 2.2. Differentially Expressed Proteins

Hereon, we defined proteins with expression level fold changes >2.0 or <0.5 with *p*-values < 0.05 as differentially expressed proteins (DEPs). Changes in the protein profile were analyzed, and 312 proteins exhibited a difference. Compared with the prehibernation group, 86 proteins were increased by more than 2-fold, and 226 proteins decreased to less than 0.5-fold during the breeding period. The detailed information of differential expressed proteins was shown in Table S3.

### 2.3. Gene Ontology Annotation and Classification Profiles

**Figure 2:**
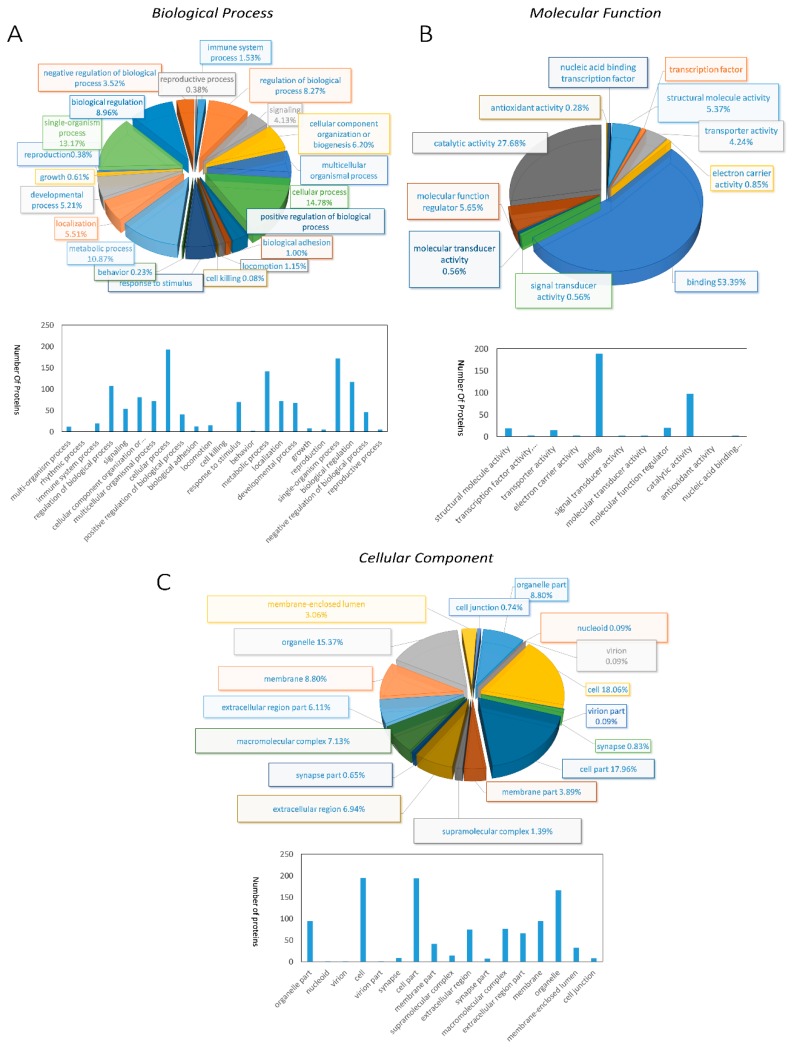
Pie diagrams and bar diagrams for gene ontology analysis of differentially expressed proteins between breeding period group and prehibernation group: (**A**) biological process, (**B**) molecular function, (**C**) cellular component.

With annotating those 312 DEPs into Gene Ontology (GO) database, we found that 240 DEPs were classified into biological process, 245 DEPs were classified into molecular function, 244 DEPs were classified into cellular component (Table S4 in Supplementary Material). Further analysis of DEPs clustered into biological processes disclosed that 193 DEPs were related to cellular process, single-organism process (172 DEPs) second, and the next were metabolic process (142 DEPs), biological regulation (117 DEPs), and regulation of biological process (108 DEPs) (Figure 2A). In the molecular function categories, DEPs were primarily associated with binding, indicating that proteins related in binding function will experience significant changes in the breeding period (Figure 2B). Cellular component ontology annotation showed that cell and cell parts possessed two-thirds of the whole DEPs, followed by organelles (Figure 2C).

### 2.4. KEGG Pathway Annotation and Reasonable Enrichment Analysis

By annotating the 312 DEPs into KEGG database, we found 177 pathways (Table S5 in Supplementary Material). The top 10 pathways were pathways in cancer, RNA transport, spliceosome, focal adhesion, PI3K–Akt signaling pathway, Huntington’s disease, Alzheimer’s disease, metabolism, extracellular matrix (ECM)–receptor interaction, and amoebiasis (Table 1).

KEGG can be used for systematic analysis of gene functions, which is linked with genomic information combined with information stored in the pathway database. The KEGG pathway is an aggregate of pathway maps showing the present knowledge on the molecular interaction networks. In our research, a database based on transcriptome data was used for identification of *Rana* oviduct proteins, which originated from different species. As a result, it is arduous for these DEPs to process KEGG pathway enrichment analysis, because the proteins were slightly different in various species. The distribution of differentially expressed proteins in various species is shown in Table 2. In the present study, the largest protein group (163 proteins, 52%) and the second largest one (61 proteins, 20%) of total DEPs was from *Xenopus tropicalis* and *Xenopus laevis*, respectively. To discover the possible signaling pathways underlying the oviduct of *Rana chensinensis*, the largest two groups of DEPs were used to process KEGG pathway analysis through KOBAS separately [11,12]. The result of KEGG enrichment was shown in Table S6. After enrichment analysis, 163 DEPs from *Xenopus tropicalis* were involved in 6 pathway terms, which were lysosome, RNA transport, glycosaminoglycan degradation, ECM–receptor interaction, metabolic pathways, and focal adhesion (Table 3). While 61 DEPs from *Xenopus laevis* were input into KEGG database for enrichment analysis, only one pathway term, pyruvate metabolism, was obtained (Table 3). Hence, KEGG enrichment for DEPs from *Xenopus tropicalis* and *Xenopus laevis* were selected to logically interpret physiological changes between prehibernation period and breeding period.

## 3. Discussion

### 3.1. Changes in Proteins Associated with Focal Adhesion and Extracellular Martix

There are three differential expressed proteins enriched in ECM (extracellular matrix)–receptor interaction pathway through KEGG enrichment analysis, collagen alpha-1 (IV) chain (COL4A1), laminin subunit alpha 2 (LAMA2), and laminin subunit beta 2 (LAMB2) (Table 3). These different kinds of ECM and ECM-associated proteins, which bind to cells through ECM receptor, play an important role in building the complex meshwork of extracellular matrices [13]. Therefore, the assembled manner of these macromolecules from ECM, including collagens, proteoglycans, laminins, and fibronectin, determine the structure and function of cell and tissue [14]. Furthermore, the resultant ECM will facilitate the biological function of a specific tissue. According to previous study, we know that the differential expression of extracellular matrix (ECM) proteins in tissue-specific cells will perform different biological processes and molecular functions [15]. Compared with the prehibernation group, COL4A1 protein in oviduct during the breeding period was upregulated with a 5-fold change. Type IV collagen, along with related other ECM proteins, is widely distributed in the basement membrane of various specific tissues [16]. Similarly, the membrane protein relevant to the morphology of oviduct was changed significantly across the estrous cycle through the bovine oviduct proteome [17]. The morphology of the oviduct muscle will be changed in *COL4A1* mutants, which then causes severe myopathy with centronuclear myofibers. The lack of biological function because of *COL4A1* mutants will lead to the gradual development of female sterility [18]. Accordingly, COL4A1 protein plays a vital role in maintaining the structure and function of the oviduct muscle. In our research, the expression of COL4A1 protein increases significantly during breeding period. Therefore, the upregulation of COL4A1 protein in breeding period may be one of notable key factors for *Rana chensinensis* to allow the oviduct to maintain optimal morphology for ovulation. 

Laminins are heterotrimeric molecules consisting of three subunit chains, named α, β, γ chain, and they significantly influence the biological processes related to cell adhesion, growth, morphology, differentiation, and migration [19,20]. There are another two laminin proteins (LAMA2, LAMB2) enriched in the ECM–receptor interaction pathway. Laminin, as a ubiquitous connective glycoprotein, is still also the predominant component of basement membrane in a specific tissue [21]. In a previous study, we know that laminin is the major component of the basement membrane in the oviduct of Japanese quail [22]. The oviduct is a key site for reproductive events that involve gamete maturation, fertilization, and early embryo development, processes that finally determine breeding success. The upregulation of LAMA2 and LAMB2, as important ECM components, could have a positive influence on the reproduction of *Rana chensinensis*. In this study, these two upregulated proteins during breeding period are supposed to be involved in preparing the oviduct for breeding time.

The second highly enriched pathway is focal adhesion, which involves four differentially expressed proteins. Focal adhesions are integrin-containing, multiplex protein structures that form mechanical connections between intracellular actin bundles and the extracellular matrix components in various cell types [23]. Besides three differentially expressed proteins from ECM–receptor interaction pathway, α-actinin (ACTN1) is enriched in the focal adhesion pathway. A previous research study has shown that mating will change the expression of α-actinin in *Drosophila* oviduct [24]. The oviduct muscles are visceral muscles that are supercontractile, meaning they have Z-bands with perforations that enlarge during contraction. The α-actinin is specific to the Z-band with perforation of supercontractile muscle, in which it crosslinks actin filaments from adjacent sarcomeres [25]. With mating of *Drosophila*, the abundance of the muscle protein α-actinin will increase markedly in the oviduct. The results in our research are consistent with this finding. By comparison with prehibernation group, the expression of α-actinin in *Rana chensinensis* oviduct is significantly upregulated during breeding time. Hence, we get the conclusion that upregulation of α-actinin (ACTN1) will probably play a vital role in maintaining reproductive function of the oviduct after hibernation.

### 3.2. Enzymes Involved in Energy Metabolism Pathway

Isocitrate dehydrogenase (NAD) subunit beta is an enzyme that, in humans, is encoded by the *IDH3B* gene. As an isocitrate dehydrogenase, IDH3 catalyzes the reversible oxidative decarboxylation of isocitrate to form α-ketoglutarate (α-KG) and CO_2_ as part of the tricarboxylic acid (TCA) cycle in glucose metabolism [26]. This step also participates in the reduction of NAD+ to NADH, which is then applied to produce ATP through the electron transport chain. Notably, IDH3 acts as NAD-specific electron acceptor, in contrast to NADP-dependent IDH1 and IDH2 [27,28]. IDH3 activity is regulated by the energy demands of the cell [27,29]. When the energy metabolism of cells is under insufficiency, IDH3 is activated by ADP. Conversely, IDH3 is inhibited by ATP and NADH under sufficiency. According to Karl’s research [30], ATP consuming processes during the breeding period could be compensated through the upregulation of ATP production. Our research has found that isocitrate dehydrogenase (IDH3B) was significantly upregulated in the breeding period, which is a method of upregulation of ATP production. The previous study also showed that the expression of isocitrate dehydrogenase (IDH) was upregulated in oviduct fluid from ewes during estrus [31]. The energy metabolism of *Rana chensinensis* oviduct, as an important site for fertilization, will involve the regulation of energy substrate and related enzymes during breeding period [32].

In this research, one another upregulated protein in energy metabolism is ubiquinone biosynthesis *O*-methyltransferase (COQ3) during the breeding period. This *O*-methyltransferase (COQ3) played a part in two steps of the reactions in the biosynthesis of ubiquinone (coenzyme Q). This enzyme methylated an early coenzyme Q intermediate, 3,4-dihydroxy-5-polyprenylbenzoic acid, as well as the final intermediate in the pathway, converting demethylated ubiquinone to coenzyme Q [33]. It is a component of the electron transport chain, and participates in aerobic cellular respiration, which generates energy in the form of ATP [34]. The complex process of fertilization required energy, which is produced by mitochondria mostly via oxidative phosphorylation [34,35]. To compensate for excess energy consumption during the breeding period, the efficiency of electron transport chain was improved through the upregulation of *O*-methyltransferase.

Guanidinoacetate *N*-methyltransferase (GAMT) is an enzyme that converts guanidinoacetate to creatine, and is encoded by the gene *GAMT*. Creatine is involved in recycling of adenosine triphosphate (ATP), the energy currency of the cell, primarily in muscle and brain tissue. This is achieved by recycling adenosine diphosphate (ADP) to adenosine triphosphate ATP via donation of phosphate groups [36]. Maternal creatine homeostasis was influenced by the expression of creatine biosynthesis-related enzymes. In a previous study, the expression of GAMT protein in heart and brain tissue was decreased in pregnant spiny mouse [37]. The finding in our research showed that the expression of GAMT was also downregulated in breeding period. Without feeding during the hibernation and breeding periods, l-arginine and glycine, as staring material of creatine biosynthesis, were inevitably at low levels in *Rana chensinensis*. Hence, the expression of GAMT was down-regulated due to deficiency of starting material. Accordingly, this result indicated that creatine biosynthesis pathway was switched off.

Adenylate kinase 7 (AK7) is a member of the adenylate kinase family of enzymes, which catalyzes the reversible phosphorylation of adenine nucleotides. It has been proven that adenylate kinase (AK) isoforms have an essential role in ciliary function and energy homeostasis [38], and mutations of *AK7* in the mouse result in primary ciliary dyskinesia [39]. The tissue specificity of Ak7, which is abundant in trachea and oviduct, has been found by Fernandez-Gonzalez et al. [39]. The expression profile of genes related to the ciliary function of the oviduct have been analyzed in porcine oviduct transcriptome [40]. It has reported that differentially expressed genes (DEGs) of porcine oviduct were enriched in GO term “cell motion” in preovulatory phase [40]. Our research has shown that AK7 expression was upregulated in oviduct during the breeding period. It is known that eggs in the ovary of *Rana chensinensis* undergo the last stages of oogenesis, and are then released into the body cavity. Finally, they are driven into the oviduct by ciliary action. In order to maintain ciliary action, AK7 provided an efficient way to transport energy from ATP production sites to the ciliary of oviducts. Hence, upregulation of AK7 will help eggs of *Rana chensinensis* to be transferred into oviduct through ciliary movement.

### 3.3. Enzymes Changed in Pyruvate Metabolism Pathway

There are three differentially expressed proteins enriched in pyruvate metabolism pathway. In contrast to prehibernation period, the expression level of pyruvate carboxylase (PC.1) and lactate dehydrogenase A (LDHA.S) during breeding period were upregulated and acetyl-CoA synthase (ACSS2.2) was downregulated. The environmental hypoxia during hibernation period contributed to metabolic acidosis in amphibians, and increased lactate concentrations in amphibian tissues [41,42]. Hence, lactate levels during breeding time should be significantly higher in *Rana chensinensis* oviduct after long hibernation times, compared with prehibernation period. The upregulated expression of lactate dehydrogenase A (LDHA) in breeding time will catalyze the transformation from lactate to pyruvic acid when environmental stress in hibernation period is relieved [43]. Moreover, the upregulation of pyruvate carboxylase will increase the production of oxaloacetate (OAA), which can be converted to phosphoenolpyruvate in gluconeogenesis, or directly enter into the TCA cycle [44]. Without eating in hibernation and breeding period, the gluconeogenesis pathway will consume the accumulation of lactate in hibernation period to reduce metabolic acidosis and increase adipose [45,46]. Accordingly, we predict that the gluconeogenesis pathway is potentially at an active stage in breeding time to decrease lactate levels in oviduct tissue. Acetyl-CoA synthetase, which is downregulated in breeding time, plays key roles in the production of acetyl-CoA from free acetate for the synthesis of fatty acid [47]. The expression of acetyl-CoA synthetase drops down when animals are starved, which leads to a decline in fatty acid synthesis [48]. While animals was refed with high carbohydrate diet, the upregulated expression of acetyl-CoA synthetase will increase fatty acid synthesis [48]. Due to no eating during the whole breeding time, the deficiency of carbon source could lead to the downregulation of acetyl-CoA synthetase. 

### 3.4. Down-Regulation of Lipid Metabolism during Breeding Period

Lipin-1, encoded by the *LPIN1* gene, possesses phosphatidate phosphatase activity [49]. In the mouse, the expression of lipin-1 is at high levels in adipose tissue and skeletal muscle, consistent with functions in lipid metabolism in these specific tissues. Actually, adipocytes in lipin-1-deficient mice fail to accumulate triacylglycerol (TAG), and do not develop mature adipocyte function [50]. In this study, the expression of lipin-1 was downregulated in the oviduct tissue during the breeding period. Accordingly, the accumulation of triacylglycerol significantly decreased. As a result, the morphology of oviduct appeared to be in a shriveled state, due to this reason. Another protein related with lipid metabolism, calcium-independent phospholipase A2 (PLA2G6), is also downregulated. PLA2 enzyme is involved in phospholipid metabolism, which is very important for many biological processes, including resembling cell membrane during cell cycle [51]. PLA2G6 helps to regulate the levels of phosphatidylcholine, which is a major membrane phospholipid [52]. Downregulation of PLA2G6 expression inhibited cell proliferation in culture, and tumorigenicity of ovarian cancer cell lines in nude mice [53]. Another reason for the shriveled state of the oviduct is that cell proliferation in *Rana chensinensis* oviduct was suppressed by the downregulation of PLA2G6 in the breeding period. 

Acetyl-CoA acetyltransferase 2 (ACAT2), an enzyme converting cholesterol and fatty acid to cholesteryl esters, is involved in lipid metabolism [54]. The previous study shows that cholesterol and fatty acid stabilize ACAT2, which is ubiquitylated on cystine residue to degrade [55]. Lipids including cholesterol and fatty acid induce the generation of reactive oxygen species, which oxidize Cys277 of ACAT2, and subsequently prevent ACAT2 from degradation [55,56]. A low level of lipid induced ubiquitylation on Cys277 for degradation of ACAT2. Accordingly, the downregulation of ACAT2 protein in this study indicated that the concentration of sterol and fatty acid was at a relatively low level in the breeding period.

### 3.5. Expression of 5-Aminolevulinate Synthase (ALAS2) in Heme Biosynthesis

The expression of 5-aminolevulinate synthase (ALAS2) was upregulated during the breeding period. ALAS is the rate-limiting enzyme in protoporphyrin IX (PPIX) production, which is the final intermediate in the heme biosynthetic pathway. Transcription of ALAS2, unlike ALAS1, is regulated by erythroid-specific transcription factors, such as GATA1 [57]. Regarding the post-transcription of ALAS2, it is affected by the content of iron. In the absence of iron, the iron-free form of iron regulatory protein (IRP) binds to iron regulatory element (IRE), forming an IRE–IRP complex that prevents translation of ALAS2 [58]. Heme, also as a downregulation factor, inhibits the translation of ALAS2 and the import of ALAS2 precursor into mitochondria [59]. According to our results about the expression of ALAS2, we concluded that the content of heme in oviduct tissue is at low levels after hibernation. Otherwise, the expression of ALAS2 was inhibited by the adequate heme. 

## 4. Materials and Methods 

### 4.1. Animals and Treatment Procedure

In this study, we performed iTRAQ proteomic analysis to identify differentially expressed proteins from the oviduct of female *Rana chensinensis* between the breeding period and the prehibernation period. Sixty adult female *Rana chensinensis* were obtained in April (*n* = 30, the breeding period), and October (*n* = 30, the prehibernation period), 2017 from Jilin Province (125°16′57″ E~131°19′12″ E, 40°51′55″ N~44°38′54″ N), China. All animals were treated in strict accordance with the recommendations in the Guide for the International Cooperation Committee Animal Welfare (ICCAW). All experimental procedures were approved by the Committee on the Ethics of Animal Experiments of Changchun University of Chinese Medicine. Frogs were anesthetized by diethyl ether. Each pair of oviducts was collected from *Rana chensinensis*.

### 4.2. Protein Extraction, Digestion, and iTRAQ Labeling

Each sample of 20 mg were frozen in liquid nitrogen and ground with a mortar and pestle. One milliliter of 10% TCA/acetone (*v*/*v*, 1:9) was added to the powder, and mixed by vortexing. The mixture was placed at −20 °C for 4 h, and then the precipitate was washed with acetone at 4 °C, until colorless. The resulting pellet was resolubilized in STD buffer (4% SDS, 100 mM DTT, 150 mM Tris-HCl, pH 8.0). The protein concentration was measured by the BCA Protein Assay kit (Beyotime, Hangzhou, China). Proteins were digested according to the FASP method [60]. The resulting mixture of each sample was labeled using iTRAQ reagent according to the manufacturer’s instructions (Applied Biosystems, Branchburg, NJ, USA). The samples of *Rana dybowskii* oviduct in prehibernation period were labeled with iTRAQ tag 113 (A) and 114 (B), and samples from the breeding period were labeled with iTRAQ tag 115 (C) and 116 (D).

### 4.3. Peptide Fractionation with Strong Cation Exchange (SCX) Chromatography

The peptide mixture was constituted again and acidified with buffer A (10 mM KH_2_PO_4_ in 25% of ACN, pH 3.0), and loaded onto a polysulfethyl column (5 µm, 4.6 × 100 mm, 200 Å, PolyLC Inc., Columbia, MD, USA). The peptides were fractionated at a flow rate of 1 mL/min with a gradient of 0–10% buffer B (500 mM KCl, 10 mM KH_2_PO_4_ in 25% of ACN, pH 3.0) for 30 min, 10–60% buffer B during 30–50 min, 60–100% buffer B during 50–55 min, 100% buffer B during 55–60 min, and finally, buffer B was set to 0% after 60 min. The detector was set at 214 nm, and fractions were collected every 1 min. The collected fractions were desalted on C18 Cartridges (Empore™ SPE Cartridges C18, Sigma, St. Louis, MO, USA) and concentrated by vacuum centrifugation.

### 4.4. LC-MS/MS Analysis

Experiments were performed as described previously [61] using a Q Exactive mass spectrometer coupled to Easy nLC (Thermo Fisher Scientific, Waltham, MA, USA). Ten microliters of each fraction was injected for nano LC-MS/MS analysis. The peptide mixture (5 μg) was loaded onto a the C18-reversed phase column (3μm, 75 μm × 10 cm) in buffer A (0.1% formic acid) and separated with a linear gradient of buffer B (80% ACN and 0.1% formic acid) at a flow rate of 250 nL/min controlled by IntelliFlow technology over 140 min. MS data was acquired using a data-dependent top10 method dynamically. The duration of dynamic exclusion was 60 s. Survey scans were acquired at a resolution of 70,000 at *m*/*z* 200 and the resolution for HCD spectra was set to 17,500 at *m*/*z* 200. Normalized collision energy was 30 eV and the underfill ratio, which means the minimum percentage of the target value at maximum fill time, was set to 0.1%. The instrument was run with peptide recognition mode enabled.

### 4.5. Protein Identification and Data Analysis

In our previous study, the transcriptome of OR was sequenced, and the corresponding unigenes were generated [62]. In the present study, the amino acid sequences translated from the CDS of unigenes were used as the protein database. The raw files were analyzed using the Proteome Discoverer 1.3 software (Thermo Electron, San Jose, CA, USA). A search for the fragmentation spectra was performed using the MASCOT search engine [63]. The results were filtered based on a false discovery rate (FDR) of no more than 0.01. The protein identification was supported by at least one unique peptide. Isobaric Labeling Multiple File Distiller and Identified Protein iTRAQ Statistic Builder were applied to calculate the ratios of protein, in which REF was used as the reference, based on the weighted average of the intensities of report ions in each identified peptide [64]. The final ratios were then normalized with the median average protein ratio, assuming that most proteins remained unchanged in abundance. Protein ratios represent the median of the unique peptides of the protein [65]. For statistical analysis, two-way ANOVA was performed for each protein, and Student’s *t*-test was used to evaluate the significant differences. The differentially expressed proteins ratio meets the fold change (≥2.0 or ≤0.5, at *p* < 0.05).

### 4.6. Bioinformatics Analysis of Differentially Expressed Proteins

We carried out gene ontology (GO) annotation analysis on the differentially expressed proteins to catalog the molecular functions, biological processes, and cellular components [66]. Mapping the function of Blast2GO (Version 3.3.5) was applied to determine GO terms correlated with DEPs [67]. To further explore the interaction of differentially expressed protein physiological process in the body and discover relationships between DEPs, KEGG enrichment analysis was performed based on KOBAS server (Version 3.0) [11,12]. KEGG pathway enrichment analyses were applied based on the Fisher’ exact test, considering the whole quantified protein annotations as background dataset. Only functional categories and pathways with *p*-values under a threshold of 0.05 were considered as significant.

## Figures and Tables

**Table 1:** Top 10 pathway terms of differentially expressed proteins (DEPs) in KEGG annotation.

Pathway Terms ^1^	DEPs with KEGG Annotation ^2^	DEPs with KEGG Enrichment ^3^
Pathways in cancer	9	0
RNA transport	8	4
Spliceosome	8	0
Focal adhesion	7	4
PI3K–Akt signaling	7	0
Huntington’s disease	7	0
Alzheimer’s disease	6	0
Metabolic pathways	24	11
ECM–receptor interaction	6	3
Amoebiasis	6	0

^1^ Pathway terms in KEGG database; ^2^ The number of differentially expressed proteins annotated in KEGG database; ^3^ The number of differentially expressed proteins enriched in top 10 pathway terms.

**Table 2:** The distribution of DEPs in different species.

Organism ^1^	Number of Proteins ^2^	Organism	Number of Proteins
*Xenopus tropicalis*	163	*Salmo salar*	2
*Xenopus laevis*	61	*Bos taurus*	2
*Rana catesbeiana*	13	*Oryctolagus cuniculus*	1
*Taeniopygia guttata*	11	*Mus musculus*	1
*Gallus gallus*	8	*Ovis aries*	1
*Monodelphis domestica*	7	*Zonotrichia albicollis*	1
*Homo sapiens*	5	*Pan troglodytes*	1
*Rana esculenta*	4	*Pyrrhocoris apterus*	1
*Mus musculus*	4	*Canis familiaris*	1
*Ailuropoda melanoleuca*	4	*Oryzias latipes*	1
*Ornithorhynchus anatinus*	3	*Equus caballus*	1
*Danio rerio*	3	*Trepomonas agilis*	1
*Rattus norvegicus*	3	*Anoplopoma fimbria*	1
*Macaca mulatta*	2	*Helicoverpa zea*	1
*Bufo japonicus*	2	*Sus scrofa*	1
*Callithrix jacchus*	2		

^1^ Organism to which protein belongs; ^2^ The number of differentially expressed proteins originates from one species.

**Table 3:** KEGG enrichment analysis of DEPs.

Pathway Terms ^1^	Input Number ^2^	Background Number ^3^	Gene Symbol of Input DEPs ^4^	*p*-Value
Lysosome	5	123	acp2, lamp1, lgmn, hexb, LOC100496969	0.0049
RNA transport	4	140	thoc6, eif3a, eif4a2, eif4b	0.0292
Glycosaminoglycan degradation	2	18	LOC100496969, hexb	0.0292
ECM–receptor interaction	3	75	lama2, lamb2, col4a1	0.0292
Metabolic pathways	11	1163	idh3b, rpn2, coq3, pla2g6, lpin1, ak7, alas2, gamt, hexb, LOC100496969, acat2	0.0292
Pyruvate metabolism	3	43	acss2.2.L, pc.1.L, ldha.S	0.0338
Focal adhesion	4	191	lama2, lamb2, col4a1, actn1	0.0451

^1^ Pathway terms in KEGG database; ^2^ The number of differentially expressed proteins enriched in one of pathway terms in KEGG database; ^3^ The total number of differentially expressed proteins in one of pathway terms in KEGG database; ^4^ The corresponding gene symbol of differentially expressed proteins enriched in one of pathway terms in KEGG database.

## Supplementary Materials

The following are available online.
